# Lysophosphatidic acid is associated with oocyte maturation by enhancing autophagy via PI3K-AKT-mTOR signaling pathway in granulosa cells

**DOI:** 10.1186/s13048-023-01228-9

**Published:** 2023-07-11

**Authors:** Jia Liu, Chong Wang

**Affiliations:** 1 Department of Otolaryngology, Children’s Hospital, Zhejiang University School of Medicine, National Clinical Research Center for Child Health, Hangzhou, 310051 People’s Republic of China; 2 Reproductive Medicine Center, Hangzhou Women’s Hospital (Hangzhou Maternity and Child Health Care Hospital), Shangcheng District, No. 369 Kunpeng Road, Hangzhou, 310008 People’s Republic of China

**Keywords:** Lysophosphatidic acid, In vitro maturation, Granulosa cells, LY294002, Small interfering RNA, Autophagy

## Abstract

**Background:**

Folliculogenesis is a complex network of interacting cellular signals between somatic cells and oocytes. Many components in ovarian follicular fluid (FF) dynamically change during folliculogenesis and play a positive role in oocyte maturation. Previous studies have reported that lysophosphatidic acid (LPA) promotes cumulus cell expansion, oocyte nuclear maturation, and in vitro maturation of oocytes.

**Results:**

Initially, the expression of LPA was raised in matured FF significantly (*P* < 0.0001). Then, 10 μM LPA treated for 24 h in human granulosa cells (KGNs) aggravated cell proliferation, with increased autophagy, and reduced apoptosis. Meanwhile, we demonstrated that LPA mediated cell function through the PI3K-AKT-mTOR signaling pathway as PI3K inhibitor (LY294002) significantly prevented LPA-induced AKT, mTOR phosphorylation and autophagy activation. Such results were also verified by immunofluorescence staining and flow cytometry. In addition, an autophagy inhibitor 3 methyladenine (3MA) could also alleviate the effects of LPA, by activating apoptosis through PI3K-AKT-mTOR pathways. Finally, we found blockade with Ki16425 or knockdown LPAR1, alleviated LPA mediated autophagy activation in KGNs, suggesting that LPA enhances autophagy through activation of the LPAR1 and PI3K-AKT-mTOR signaling pathways.

**Conclusion:**

This study demonstrates that increased LPA activated PI3K-Akt-mTOR pathway through LPAR1 in granulosa cells, suppressing apoptosis by enhancing autophagy, which might play a role in oocyte maturation in vivo.

**Supplementary Information:**

The online version contains supplementary material available at 10.1186/s13048-023-01228-9.

## Background

During the folliculogenesis, oocytes are always wrapped by granulosa cells (GCs) and exist in the follicular cavity within ovarian microcirculation. Many studies have confirmed that follicular fluid (FF) and cumulus granulosa cells play an important role in the regulation of oocyte development and fertilization [1, 2]. Many components in FF dynamically change during folliculogenesis and play a positive role in oocyte maturation [3–5].

LPA is a substance that is enriched in FF, and also the smallest and simplest phospholipid (430–480 Da) found so far. It is a key precursor in the early stage of phospholipid biosynthesis in eukaryotic cells. LPA is divided into glycerol backbone, fatty acyl chain and phosphate group [6]. LPA can be synthesized both in the cell as an intermediate of phospholipid synthesis and extracellular as an intercellular signal molecule [7], thus exerting a wide range of biological effects. It has been proved that LPA can improve the in vitro maturation (IVM) rate of human oocytes [8], as well as that of golden hamster [9], mouse [10] and bovine [11].

Hinokio et al. [9] found that the stimulation effect of LPA on the maturation of golden hamster oocytes must be exerted by cumulus granulosa cells, which means no positive effect on naked eggs. Kuwahara et al. [12] proved that LPA in mouse FF can participate in cumulus granulosa cell expansion before ovulation by stimulating hyaluronan production. The latest research of He et al. [13] reveals the regulation of selective autophagy of pig/human cumulus granulosa cells on the maturation quality and fertilization ability of oocytes through co-culture of GCs and oocytes. However, knowledge about the role of LPA on the progression of GCs and oocyte maturation is largely unknown.

LPA act as extracellular signaling molecules by binding to and activating G protein-coupled receptors (GPCRs), which exist as multiple subtypes (LPAR1-6) [14]. LPAR1-3 belongs to the endothelial cell differentiation gene family, and LPAR4-6 belongs to the purine receptor family. To our knowledge, LPA can activate various downstream signaling pathways involving ERK1/2, MAPK, uPA-uPAR, Ras, and AKT [15–17]. Activation of those pathways can drive central cell process, including proliferation, migration, differentiation, apoptosis, and autophagy. High level of LPA can activate PI3K-AKT-mTOR pathway through LPAR, leading to cascade reaction and affecting the metastasis and invasion of ovarian cancer [18]. LPA also inhibits cisplatin-induced apoptosis by activating PI3K-AKT-mTOR signaling pathway in Hela cells [19].

In the present study, the expression level of LPA in the FF during oocyte maturation, along with the associated underlying mechanism of GCs, were investigated.

## Results

### LPA is up-regulated in matured oocytes in vivo

**Fig. 1:**
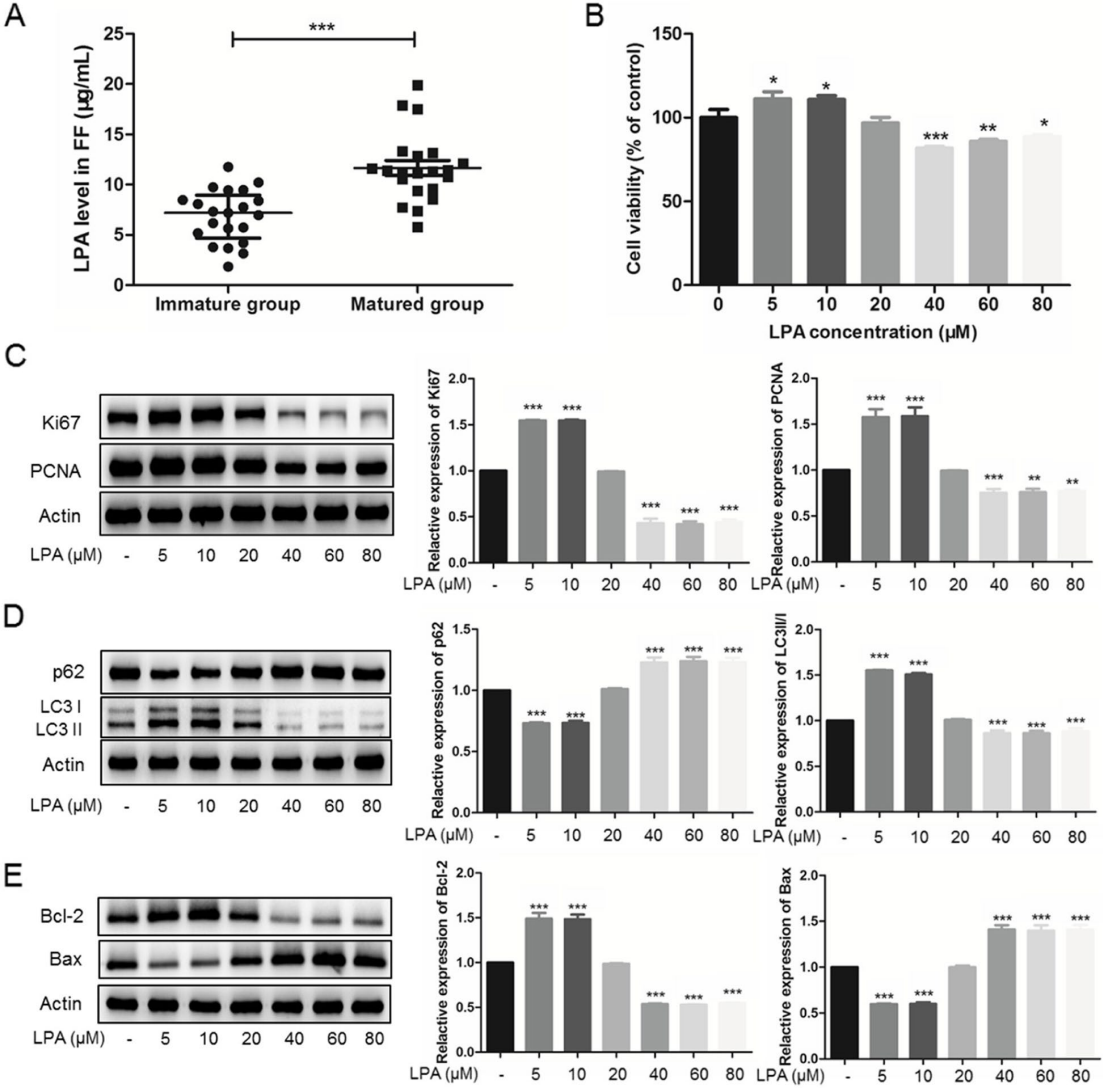
Over-expressed LPA regulates follicular microenvironment in vivo and in vitro*.* **A** Enzyme-linked immunosorbent assay result of LPA in follicular fluid of matured and immature oocytes (*n* = 21). (B) Cell counting kit-8 assay results of KGNs treated with different concentrations of LPA for 24 h. The protein levels of Ki67, PCNA, p62, LC3 II/I, Bcl-2, and Bax were detected by Western Blotting under different concentrations of LPA treatment (C-E, cropped gels). *n* = 3 per group in (B)-(E). Data are presented as means ± SEM; ^*^P < 0.05, ^**^P < 0.01, ^***^P < 0.0001

We recruited a total of 21 infertile patients with both mature and immature oocytes, and also collected serum on the hCG trigger day. The age of the collected patients is 30.32 ± 2.32 years old; the infertility duration is 2.46 ± 1.40 years; and the BMI is 21.84 ± 3.29 kg/m^2^. The serum level of LPA is 4.44 ± 1.60 μg/mL in our collected patients. Compared with immature oocytes, LPA expression level of FF was found to be significantly up-regulated in matured ones (11.65 ± 3.36 *vs.* 6.87 ± 2.55 μg/mL, *P* < 0.0001, Fig. 1A).

### LPA regulates KGNs activity including autophagy in vitro

The cell viability was determined by CCK-8 in KGNs treated with different concentrations of LPA (0, 5, 10, 20, 40, 60, and 80 μM) for 24 h. As shown in Fig. 1B, at an appropriate concentration (0–10 μM), LPA promotes KGNs proliferation. Interestingly, highly concentrations of LPA (40–80 μM) impaired KGNs proliferation activity. Same proliferation changing trend were measured using Ki67 and PCNA, two cell cycle related antigens, by Western blotting (Fig. 1C). The marker protein of autophagy and apoptosis was also determined by Western blotting in KGNs. LPA caused decreased p62 expression and a concomitant increase in LC3 II-to-I ratio at an appropriate concentration (5, 10 μM) (Fig. 1D). Meanwhile, LPA significantly increased Bcl-2 expression and decreased Bax expression under 5 and 10 μM LPA treatment (Fig. 1E). These findings indicate that LPA enhances autophagy and inhibits apoptosis in vitro with 5 and 10 μM treatment.

### LPA regulated PI3K-AKT-mTOR pathway in KGNs

**Fig. 2:**
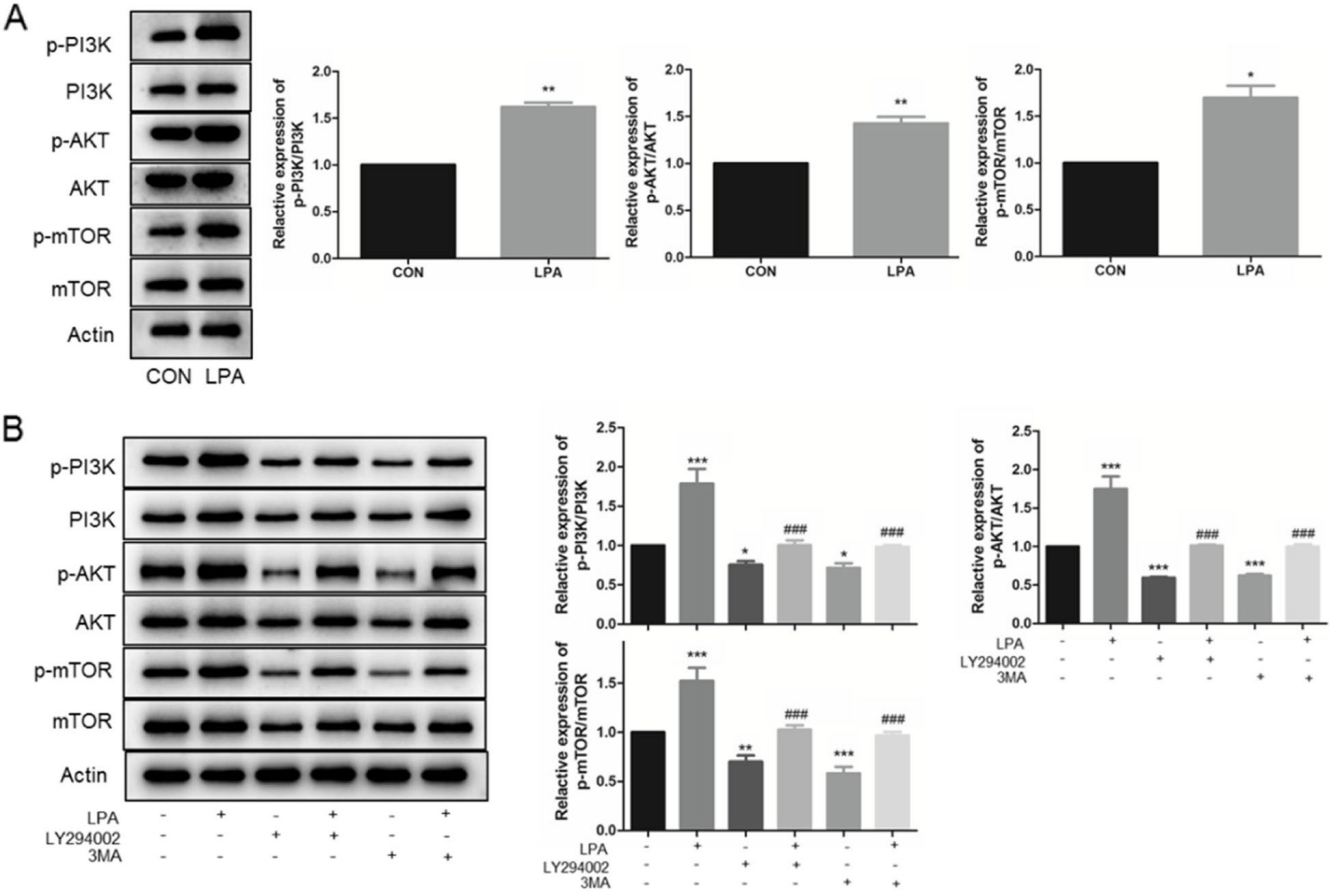
LPA regulates through the PI3K-AKT-mTOR pathway. **A** The protein levels of PI3K-AKT-mTOR pathway were detected by Western Blotting (cropped gels) under 24 h exposure of 10 μM LPA. B Serum-starved KGNs were treated with 20 μM LY294002 (PI3K inhibitor) for 24 h, as well as 5 mM 3MA (autophagy inhibitor) for 24 h, before exposure to 10 μM LPA for 24 h. The protein levels of PI3K-AKT-mTOR pathway were detected by Western Blotting (cropped gels). *n* = 3 per group. Data are presented as means ± SEM; ^*^P < 0.05, ^**^P < 0.01, ^***^P < 0.0001 *vs.* CON, ^#^*P* < 0.05, ^##^*P* < 0.01, ^###^*P* < 0.0001 *vs*. LPA

The difference of LPA expression level between matured *vs.* immatured FF was 11.65 ± 3.36 *vs.* 6.87 ± 2.55 μg/mL (Fig. 1A), converted as 25.41 ± 7.33 *vs.* 14.98 ± 5.56 μM*.* So, we chose 10 μM to explore the effect of LPA on KGNs for the follow-up study. Then, we measured PI3K-AKT-mTOR pathway associated proteins (AKT, phosphorylated (p)-AKT, PI3K, p-PI3K, mTOR and p-mTOR) to determine whether LPA might also target PI3K-AKT-mTOR pathway in KGNs. As shown in Fig. 2A, p-PI3K/PI3K, p-AKT/AKT, and p-mTOR/mTOR were all up-regulated after LPA treatment (^**^P < 0.01, ^**^P < 0.01, ^*^P < 0.05, respectively).

### PI3K-AKT-mTOR pathway is required for LPA-induced autophagy enhancing

To determine whether or not PI3K-AKT-mTOR pathway is required in LPA treatment, a PI3K inhibitor (LY294002) was used. Furthermore, to determine whether or not PI3K-AKT-mTOR pathway is required in LPA-induced autophagy, an autophagy inhibitor (3MA) was used. Importantly, we found that the positive effects of LPA in increase the phosphorylation of PI3K and its downstream factors (Akt and mTOR) were abrogated by LY294002, a PI3K inhibitor (^###^*P* < 0.0001, ^##^*P* < 0.01, Fig. 2B). The autophagy inhibitor 3MA also represented the same effect on PI3K-AKT-mTOR pathway attenuating LPA effects (^###^*P* < 0.0001, ^##^*P* < 0.01, Fig. 2B).

**Fig. 3:**
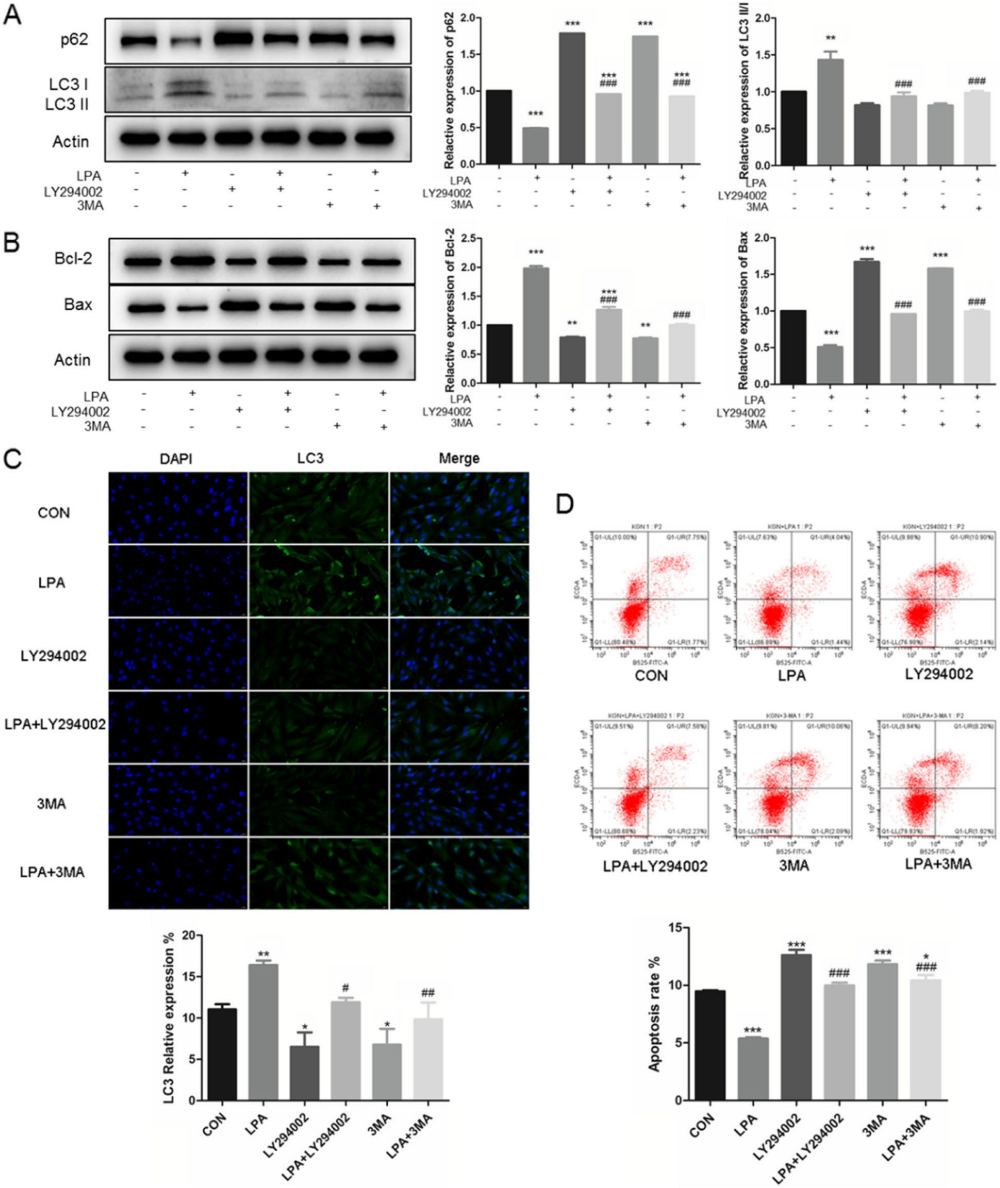
LPA up-regulates autophagy through the PI3K-AKT-mTOR pathway. Serum-starved KGNs were treated with 20 μM LY294002 (PI3K inhibitor), as well as 5 mM 3MA (autophagy inhibitor) for 24 h, before exposure to 10 μM LPA for 24 h. The autophagy related protein levels (**A**) and apoptosis related protein levels **B** were detected by Western Blotting (cropped gels). Immunofluorescence staining of LC3 **C**, and flow cytometry analysis **D** were also taken. *n* = 3 per group. Data are presented as means ± SEM; ^*^P < 0.05, ^**^P < 0.01, ^***^P < 0.0001 *vs.* CON, ^#^*P* < 0.05, ^##^*P* < 0.01, ^###^*P* < 0.0001 *vs*. LPA

In addition, the decreased LC3 II-to-I ratio, increased p62 (all ^###^*P* < 0.0001, Fig. 3A), decreased Bcl-2, and increased Bax expression (all ^###^*P* < 0.0001, Fig. 3B) were all found in LY294002 and 3MA treatment with LPA, which represents autophagy inhibition and apoptosis activation. Such results were also verified by immunofluorescence staining (Fig. 3C) and flow cytometry (Fig. 3D). Together, we found that PI3K inhibitor and autophagy inhibitors could all alleviate the effects of LPA, by activating apoptosis through PI3K-AKT-mTOR pathways. These data indicate that the PI3K-AKT-mTOR pathway is required in mediating LPA-induced autophagy enhancing.

### LPA enhance autophagy through LPAR1

**Fig. 4:**
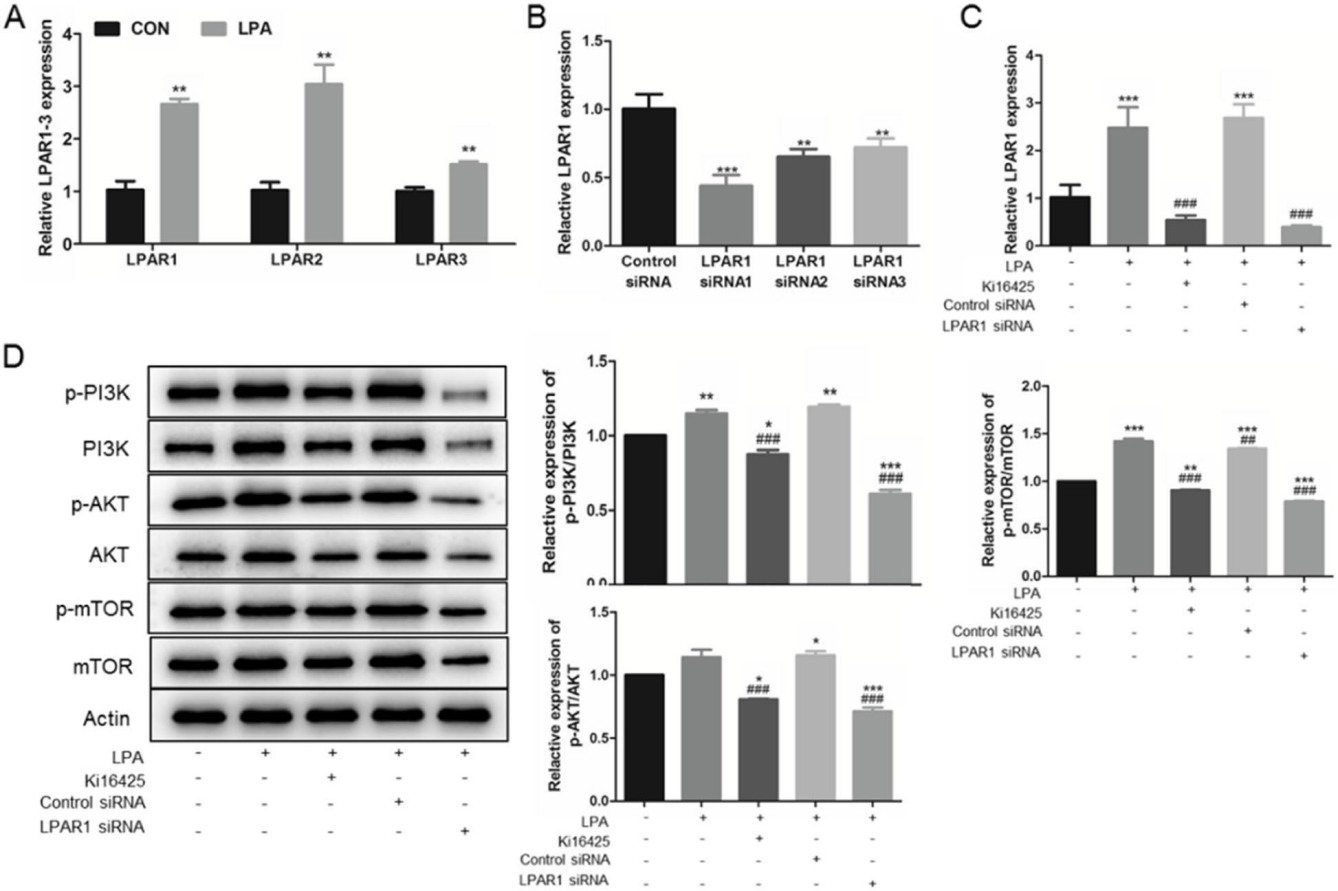
Knockdown of LPAR1 attenuates LPA-mediated PI3K-AKT-mTOR pathway activation. **A** KGNs were seeded and treated 10 μM LPA for 24 h. LPAR1, LPAR2, and LPAR3 mRNA expression was analyzed by qRT-PCR. **B** LPAR1 was knocked down by siRNA with the primer sequences list in Table 1. Serum-starved KGNs were treated with 10 μM Ki16425 (LPAR1/3 inhibitor) for 24 h, before exposure to 10 μM LPA for 24 h. Also, control siRNA and LPAR1 siRNA1 were transfected into KGNs. **C** *LPAR1* mRNA expression was verified by real-time PCR. **D** The protein levels of PI3K-AKT-mTOR pathway were detected by Western Blotting (cropped gels). *n* = 3 per group. Data are presented as means ± SEM; ^*^P < 0.05, ^**^P < 0.01, ^***^P < 0.0001 *vs.* CON, ^#^*P* < 0.05, ^##^*P* < 0.01, ^###^*P* < 0.0001 *vs*. LPA

We examined LPAR1-3 mRNA expression with LPA treatment. We found that LPAR1-3 were significantly higher than that in control cells (Fig. 4A). As, LPAR1 and LPAR3 have been proved to be related to the activation of PI3K-AKT-mTOR pathway; considering the difference degree, we chose LPAR1 for follow-up study.

**Fig. 5:**
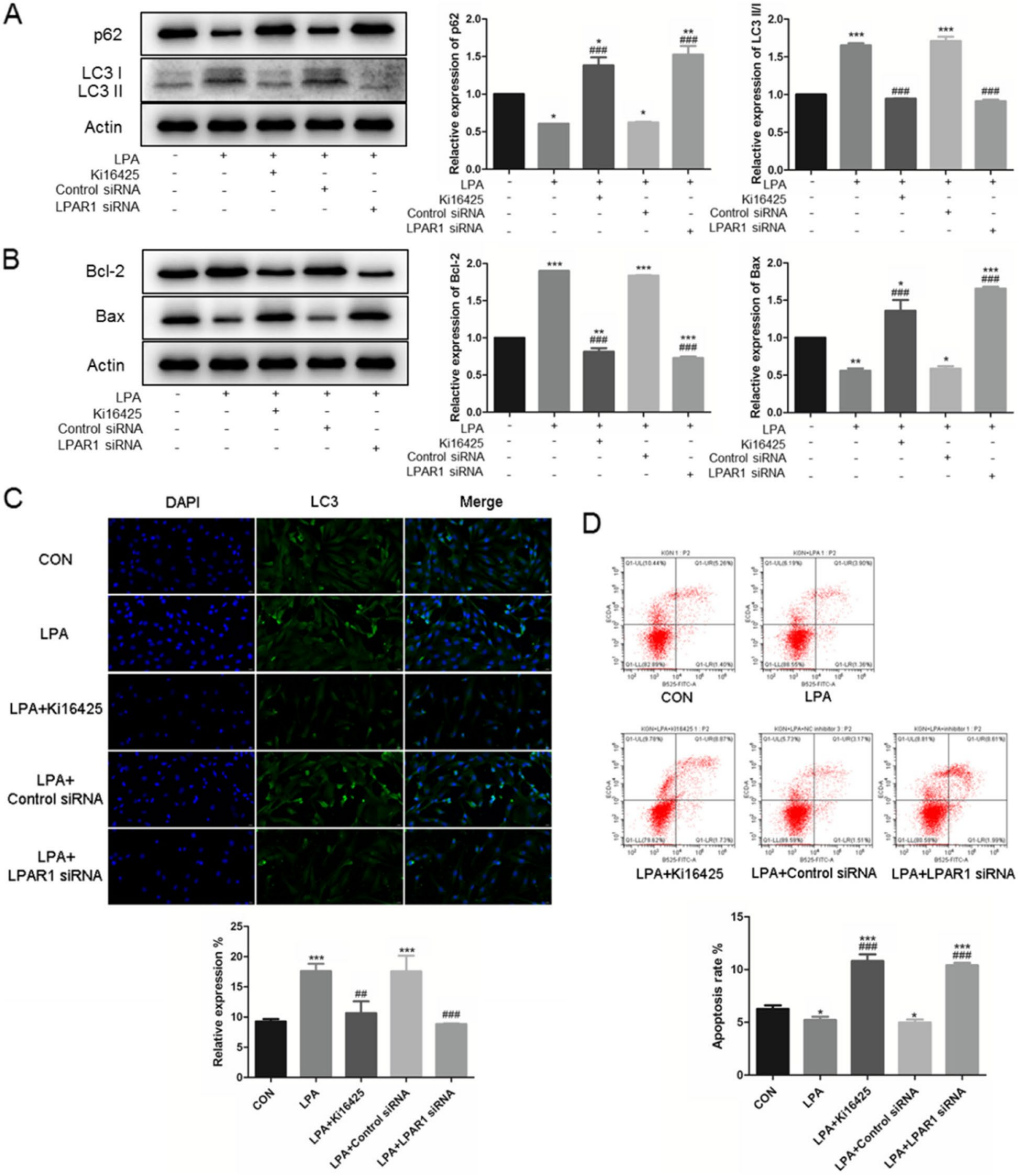
Knockdown of LPAR1 attenuates LPA-mediated autophagy enhancement. Serum-starved KGNs were treated with 10 μM Ki16425 (LPAR1/3 inhibitor) for 24 h, before exposure to 10 μM LPA for 24 h. Also, control siRNA and LPAR1 siRNA were transfected into KGNs. The autophagy related protein levels (**A**) and apoptosis related protein levels (**B**) were detected by Western Blotting (cropped gels). Immunofluorescence staining of LC3 (**C**), and flow cytometry analysis (**D**) were also taken. *n* = 3 per group. Data are presented as means ± SEM; ^*^P < 0.05, ^**^P < 0.01, ^***^P < 0.0001 *vs.* CON, ^#^*P* < 0.05, ^##^*P* < 0.01, ^###^*P* < 0.0001 *vs*. LPA

The non-selective LPAR1/3 antagonist, Ki16425, was used to determine whether LPAR1 is involved in up-regulated autophagy in KGNs by LPA. LPAR1 was also knocked down by siRNA (Figs. 4B), and *LPAR1* mRNA expression was verified by real-time PCR (Figs. 4C). In our study, we found that Ki16425 weakens the activation of PI3K-AKT-mTOR pathway induced by LPA treatment (Figs. 4D). Furthermore, it also weaken the up-regulation of autophagy caused by LPA, indicating by LC3 II-to-I ratio, p62 expression (^###^*P* < 0.0001, ^###^*P* < 0.0001, Fig. 5A), and immunofluorescence staining (Fig. 5C); as well as improving apoptosis (Figs. 5B, 5D). Moreover, knock-down of LPAR1 through siRNA transfection also reduced LPA-mediated effects through PI3K-AKT-mTOR pathways (Fig. 4D), decreasing apoptosis inhibition and autophagy enhancement (Fig. 5). Our results revealed that LPA-LPAR1 signaling activates PI3K-AKT-mTOR pathway to enhance autophagy in KGNs.

## Discussion

LPA, a phospholipid signaling molecule, is produced from circulating lysophosphatidylcholine (LPC) by autotaxin, which is involved in oocytes IVM of multiple species [8–11]. In the current study, we found (i) up-regulated LPA level in matured FF in vivo; (ii) supplemental LPA promoted cell proliferation and autophagy in KGNs, as inhibited apoptosis in vitro*;* (iii) LPA up-regulated autophagy through PI3K-AKT-mTOR signaling pathway by LPAR1.

Tokumura et al. [20]. demonstrated the LPA concentration in human FF is 10 ~ 25 μM under physiological conditions, while Yamamoto et al. [21] found the average concentration of LPA is 24.5 *vs.* 30.3 μM (small *vs.* large follicles). In the present study, LPA level increased in human FF during oocytes maturation in vivo (11.65 ± 3.36 *vs.* 6.87 ± 2.55 μg/mL, converted as 25.41 ± 7.33 *vs.* 14.98 ± 5.56 μM, Fig. 1A), consist with the former literature [21]. Meanwhile, the supplemental LPA treatment also showed that appropriate dose of LPA (5, 10 μM), similar with the dose range during oocytes maturation, promoted KGNs cell proliferation (Fig. 1B, C) in vitro. The signal transduction in LPA-induced oocytes nuclear maturation [22] is identical to the signal transduction in the LPA-stimulated cumulus expansion [12], which suggested that LPA plays an important role in oocyte maturation, even in ovulation.

We then investigated the molecules involved in autophagy (p62, LC3 II/I, Fig. 1D) and apoptosis (Bcl-2, Bax, Fig. 1E). Former investigations showed that exposure to LPA during oocyte maturation reduced the extent of apoptosis in bovine cumulus-oocyte complexes (COCs) [11]. Yuan et al. [23] also suggested the degree of apoptosis in COCs is negatively correlated with the developmental competence of the oocytes. To our knowledge, GCs are the target for LPA action [24]. Up-regulated LPA might lead to the stimulation of anti-apoptotic processes in the GCs, and also GCs differentiation and proliferation in healthy oocytes [25]. These results are consistent with our study, which is shown that LPA treatment stimulates the anti-apoptotic process of KGNs (Fig. 1E).

Previous evidence has shown that LPA improves oocyte quality by activating the ERK1/2 pathway [15]. Also, literature proved LPA increases IVM efficiency via uPA-uPAR signaling pathway in GCs [16]. To date, accumulating evidence demonstrated that PI3K pathway is activated in preimplantation embryonic development [26, 27]. Our date proved LPA promoted the phosphorylation of PI3K and its downstream targets (Fig. 2A). Furthermore, we indicate that PI3K-AKT-mTOR pathway is required for LPA-induced KGNs cell progresses, as LPA-activated signal was significantly prevented by a PI3K inhibitor (Fig. 2B).

In addition, PI3K-AKT-mTOR pathway has different interactive regulation modes of autophagy and apoptosis. Han et al. [28] demonstrated the combination treatment of curcumin and probucol enhanced autophagy and reduced apoptosis via the PI3K-Akt-mTOR pathway in chondrocytes. And, Liu et al. [29] suggested the ginsenoside-Rg5 induced cell apoptosis and autophagy in human osteosarcoma, while the inhibition of autophagy by 3MA reduced cell apoptosis. In this study, we confirmed that LPA effect on KGNs via PI3K-AKT-mTOR pathway, resulting in enhanced autophagy and reduced apoptosis. Furthermore, the inhibition of autophagy could alleviate the LPA-related apoptosis inhibition (Fig. 3).

Reports indicate that the LPA-LPAR pathway promotes the transcription and phosphorylation of molecules in PI3K-AKT-mTOR pathway, leading to autophagy and/or apoptosis regulation [30, 31]. To verify the involvement of LPAR in LPA-induced KGNs autophagy, we used ki16425 (an antagonist of LPAR1 and LPAR3) and RNA interference system. Our data provide evidence that LPA might cause autophagy promotion through LPAR1 via PI3K-AKT-mTOR pathway (Fig. 4). In addition, we found that knockdown of LPAR1 blocked LPA-regulated autophagy promotion, indicating that LPAR1 is involved in LPA-induced cell progresses (Fig. 5). Together, our data suggests that LPA-induced KGNs cell autophagy is partially promoted through LPAR1-PI3K-Akt-mTOR pathway. Since LPAR2/3 is also expressed differently, further studies are needed to clarify the role of other LPAR.

There may be some other mediators responsible for the effects of LPA, for example, prostaglandin endoperoxide synthase 2 (PTGS2) [32], amphiregulin (AREG), and epiregulin (EREG) [11, 33]. Thus, it would be interesting to know whether LPA-mediated GCs expansion and ovulation. Furthermore, animal experiments confirming PI3K-AKT-mTOR pathway for LPA treatment were also needed to be take in the future experiment.

## Conclusions

In summary, we suggest that increased LPA activated PI3K-Akt-mTOR pathway through LPAR1 to promote GCs autophagy, which might play a role in oocyte maturation in vivo. Importantly, our study suggested that LPAR1 might prompt a potential therapeutic target for diminished ovarian response patients, and LPA might be the potential addition to human IVM medium.

## Methods

### Clinical specimens

This study was approved by the Ethics Committee of the Faculty (Hangzhou Women’s Hospital, approval number: 2017–479, 2021-K2-15). Written informed consent was obtained from all subjects before collection. We recruited a total of 21 infertile patients from October 2018 to July 2020 with both mature and immature oocytes. Serum was collected on the hCG trigger day. Ovarian FF was collected from each follicle separately by oocyte retrieval operation, and allocated according to the maturity of degranulated oocytes. Immediately following oocyte retrieval, FF was collected and then centrifuged at 3000 g for 15 min to collect the supernatant. The characteristic information as age, infertility duration, BMI were also collected.

### Enzyme-linked immunosorbent assay

The human lysophosphatidic acid (LPA) ELISA kit (CSB-EQ028005HU, CUSABIO Co., Wuhan, China; detection limit, 3.9 ng/mL) was used to detect LPA level in FF and serum with dilution of 1:400. The protein concentrations were measured according to the manufacturer’s instructions.

### Cell culture and treatment

Human granulosa-like tumor cell line (KGNs, iCell-h298, icell bioscience Inc, Shanghai, China) were cultured in RPMI 1640 containing 10% fetal bovine serum and 1% penicillin/streptomycin at 37 °C with 5% CO_2_. KGNs were treated with various concentrations of LPA (0, 5, 10, 20 and 40 μM; L-7260, Sigma-Aldrich, Shanghai, China) for 24 h when performing cell counting kit-8. Then, KGNs were treated with 20 μM LY294002 (an inhibitor of PI3K; S1105, Selleck, Shanghai, China), as well as 5 mM 3 methyladenine (3MA, autophagy inhibitor; S2767, Selleck) for 24 h, before exposure to 10 μM LPA for 24 h. LPAR1 inhibitor and controls were purchased from the GenePharm (Shanghai, China), and KGNs were treated with 10 μM Ki16425 (LPAR1 and LPAR3 antagonist; S1315, Selleck) for 24 h, before exposure to 10 μM LPA for 24 h.

### Cell counting kit-8 (CCK-8)

KGN cells were seeded onto a 96-well plate at the density of 5 × 10^3^ cells/well for 24 h. Then, discard the cell culture medium. Adding 100 μL of fresh medium containing 0.5% FBS as well as 10 μL CCK-8 (C0038, Beyotime Biotechnology, Shanghai, China) into each well, and then incubated for 2 h at 37 °C. Optical density value was determined using microplate reader by 450 nm.

### Western blot analysis

Western blots were performed as previously described [5]. Cells were lysed with 200 μL of RIPA lysate buffer (P0013B, Beyotime Biotechnology) plus 1 mM PMSF at 4 °C for 30 min. After centrifugation at 12,000 rpm for 10 min at 4 °C, the supernatant was harvested and stored at -80 °C. The protein concentration was determined using BCA quantitative kit (P0012, Beyotime Biotechnology). Equal amounts of protein (10 mg) were subjected to 10% SDS-PAGE electrophoresis and transferred to a PVDF membrane (IPVH00010, Millipore, Massachusetts, USA). Further, the membranes were blocked in 5% (w/v) skimmed milk at 37 °C for 2 h, and the primary antibodies were respectively added for incubation at 4 °C overnight. Secondary antibodies as goat anti-mouse IgG-HRP (SA00001-1, Proteintech Technology, 1:1000) and goat anti-rabbit IgG-HRP (SA00001-2, Proteintech Technology, 1:1000) were then incubated with membrane at room temperature for 1.5 h. The blots were visualized using the ECL Plus Luminous Kit (S17851, Yeasen Biotechnology, Shanghai, China). At last, the results were measured with Image J software.

Some of the primary antibodies were purchased from Abcam (Massachusetts, USA), including anti p62 antibody (ab109012, 1:10,000), anti Bcl-2 antibody (ab182858, 1:2000), anti PI3K (p85 alpha) antibody (ab191606, 1:1000), antiphosphorylated mTOR (ser2448) antibody (ab109268, 1:5000), and anti mTOR antibody (ab134903, 1:10,000). Anti proliferating cell nuclear antigen (PCNA) antibody (60097-1-Ig, 1:5000), anti Bax antibody (60267-1-Ig, 1:5000), anti LC3 antibody (14600-1-AP, 1:1000), anti AKT antibody (60203-2-Ig, 1:5000), anti phospho-AKT (Ser473) antibody (66444-1-Ig, 1:2000), and anti beta-actin antibody (66009-1-Ig, 1:20,000) were purchased from Proteintech Technology (Wuhan, China). Anti p-PI3K (Tyr458) antibody (4228 T, 1:1000) was purchased from Cell Signaling Technology (Massachusetts, USA) while anti Ki67 antibody (A11390, 1:1000) was purchased from ABclonal Technology (Wuhan, China). All original gel images were represented in Supplementary file 1.

### Immunofluorescence staining

Prior to immunofluorescence staining, the KGNs in 6‑well plates were rinsed three times with PBS and then fixed with 4% paraformaldehyde for 15 min at room temperature. After rinsed three times with PBS, cells were blocked with goat serum albumin (C0265, Beyotime Biotechnology) for 60 min at room temperature. Then, incubate cells at 4˚C overnight in a solution containing LC3 antibody. Subsequently after PBST rinsing, the cells were stained with an Alexa Fluor 488‑conjugated anti‑mouse antibody (SA00006-1, Proteintech Technology) for 60 min at 25˚C. Finally, rinsed with PBST and incubated with DAPI for 5 min in dark. The images were captured by a fluorescence microscope (Olympus Corporation, Tokyo, Japan).

### Flow cytometry

Flow cytometry was detected by Annexin V-AbFluor™ 488 apoptosis kit (KTA0002, Abbkine Scientific, Shanghai, China). The cultured cells were washed with PBS to prepare single cell suspension, and the number of cells was adjusted to 1–5 × 10^6^ cells/mL. KCNs were collected by centrifugation at 1000 r/min for 5 min. Discard the supernatant, washed with PBS once, and added Annexix V to re-suspended cells. Then turn 100 μL to new flow detection tube, and added 5 μL Annexin V- AbFluor™ 488 and 2 μL PI working solution, incubated for 15 min in dark. Finally, added 400 μL Annevix V buffer, and detected in 1 h.

### RNA isolation and quantitative reverse transcription-polymerase chain reaction (qRT-PCR)

Total RNA was extracted from cultured KGNs with Trizol, and determined by a Nanodrop 2000 (ThermoFisher Scientific, MA, USA). And cDNA was generated from 1 μg RNA using Hifair® II 1st Strand cDNA Synthesis SuperMix for Qpcr (11123ES60, Yeasen Biotechnology). The Hieff® qPCR SYBR Green Master Mix (11202ES08, Yeasen Biotechnology) was used to detect and quantify the mRNA levels of each gene (ABI QuantStudio™ 12 K Flex, ThermoFisher Scientific). All specific sequence primers included in the present work were listed in Table 1.

**Table 1:** List of primer sequences used for RT-PCR

Name	Sequence
GAPDH	TCAAGAAGGTGGTGAAGCAGG
	TCAAAGGTGGAGGAGTGGGT
LPAR1	CCAGAGCTTTTCGAGTGGGA
	TAGGTGGATGGGGAGCTTCA
LPAR2	GGGGTCACGGCGGGAATTT
	TGATGACCATCTCATGCGCC
LPAR3	GCAGGCTGAATCCCTTCCAT
	CCAGGTACCAGAAGGATGCC
Control siNRA	GAACGUCACGGCUGUUAAAGCTT
	GCUUUAACAGCCGUGACGUUCTT
Human LPAR1 siRNA1	GGUCAUGGUGGCAAUCUAUGUTT
	ACAUAGAUUGCCACCAUGACCTT
Human LPAR1 siRNA2	GCAAUCGAGAGGCACAUUACGTT
	CGUAAUGUGCCUCUCGAUUGCTT
Human LPAR1 siRNA3	GGAUACCAUGAUGAGUCUUCUTT
	AGAAGACUCAUCAUGGUAUCCTT

### Transfection of siRNA into KGNs

KGNs were transfected with siRNA of LPAR1 for 24 h before stimulated with LPA for 24 h. Cells were transfected with 50 nM oligonucleotides via Lipofectamine 2000 (11668019, ThermoFisher Scientific). All the siRNA sequences were listed in Table 1.

### Statistical analysis

Data were presented as the mean ± standard deviation. Differences between two groups were tested by Student’s t-test; while oneway ANOVA was used among three or more groups. A *P* < 0.05 was considered statistically significant. All statistical analyses were performed using Graphpad Prism 5.01.

## Supplementary Information

**Additional file 1.**

## Data Availability

All data analysed during this study are included in this published article. Raw data are available from the corresponding author on reasonable request.
